# The PERSIAN Cohort: Prevalence of Psychiatric Disorders Among Employees

**DOI:** 10.34172/aim.2024.12

**Published:** 2024-02-01

**Authors:** Mostafa Farahbakhsh, Elnaz Faramarzi, Ali Fakhari, Mahshid Sadeghi, Habibeh Barzegar, Sanaz Norouzi, Sepideh Harzand-Jadidi

**Affiliations:** ^1^Research Center of Psychiatry and Behavioral Sciences, Tabriz University of Medical Sciences, Tabriz, Iran; ^2^Liver and Gastrointestinal Diseases Research Centre, Tabriz University of Medical Sciences, Tabriz, Iran; ^3^Department of Psychiatry, School of Medicine, Tabriz University of Medical Sciences, Tabriz, Iran

**Keywords:** Anxiety, Depression, Mental disorder, Obsession, Prevalence

## Abstract

**Background:**

Considering the impact of psychiatric disorders on the productivity of individuals and society’s economy, we aimed to determine the prevalence of psychiatric disorders among the employees of Tabriz University of Medical Sciences.

**Methods:**

This cross-sectional study was conducted on 1282 employees of Tabriz University of Medical Sciences in 2019. The required data were collected by trained psychologists using Composite International Diagnostic Interview (CIDI). In this process, psychiatric disorders were considered dependent variables, and demographic variables as independent variables. The relationship between independent and dependent variables was assessed using the chi-square test and Binary logistic regression in Stata version 17.

**Results:**

The prevalence of generalized anxiety disorder (GAD), major depressive disorder (MDD), and obsessive-compulsive disorder (OCD) among employees was 14.12%, 12.48%, and 3.9%, respectively. The prevalence of GAD in women was significantly higher than men (17.06% vs. 10.02%, *P*<0.001). The prevalence of GAD was 42.86%, 15.97%, 13.49%, and 16.67 in widowed, single, married, and divorced employees, respectively (*P*=0.016). The prevalence of MDD in women was significantly higher than men (16.59% vs. 7.64%, *P*<0.001). The prevalence of MDD was 16.3%, 11.2%, 9.6%, and 8.56% in employees with Bachelor’s, Associate, Master’s degree, and High school diploma, respectively (*P*=0.009).

**Conclusion:**

Considering the relatively high prevalence of GAD and MDD among the employees of Tabriz University of Medical Sciences, strengthening counseling centers in the university and encouraging employees to participate in these centers, and examining them in terms of mental health help identify people at risk of mental disorders in time and provide counseling services to these people.

## Introduction

 Psychiatric disorders are one of the common problems in the health system worldwide. These disorders are considered among the main causes of disability, such that 14% of the global burden of diseases is attributed to psychiatric disorders.^1,2^ In some high-income countries, it is estimated that 40% of disabilities are caused by psychiatric disorders.^3^ Psychiatric disorders are also common in the working population and show a growing concern due to the decrease in the productivity of employees and organizations.^4^

 Reports have introduced psychiatric disorders among the leading causes of absenteeism and death in the workplace worldwide. Psychiatric disorders in the work environment may decline employees’ efficiency through increasing job errors, poor decision-making, loss of motivation and work commitment, and tension and conflicts with colleagues.^5^ Depression is known as one of the most common psychiatric disorders in the working population.^6^ According to estimates, depression-related absenteeism costs the United States more than $30 billion annually.^7^ Major depression can severely affect an employee’s behavior, cognition, and performance. Depressed people are more likely to lose their jobs and experience burnout more than others.^8,9^ The effects of depression on work attendance, the length of absence, employees’ accuracy in performing tasks, and their efficiency have been proven. Depression leads to the poisoning of the workplace atmosphere and tension in interpersonal relationships and the work process.^10^ Epidemiological studies show that depression is one of the costliest diseases of the work population. Employees with this disorder face disability to perform tasks almost 27 times more than healthy employees.^11,12^ Job stress is another cause of job disabilities and early retirement. Almost all employees feel some degree of nervous pressure and stress in the workplace.^13^ Occupational stress can cause poor health and increase the rate of work-related injuries and accidents. Some possible causes of job stress include overwork, lack of clear instructions, unreasonable deadlines, and lack of job security.^14^ According to a study in 2005, 20% of European employees suffer from stress or work pressure and believe that work pressure has endangered their health.^15^ In a study by Wilson et al in India on health workers, the prevalence of anxiety and major depression was reported at 17.7% and 11.4%, respectively.^16^ Que et al reported the prevalence of generalized anxiety disorder (GAD) and depression to be 46% and 44.4%, respectively, among Chinese healthcare workers during the COVID-19 pandemic.^17^ According to Caplan’s findings, 47% of physicians, managers, and hospital consultants in the healthcare sector had psychiatric disorders.^18^ Garooci Farshi and Mani reported that in Tabriz, depression, obsession, and anxiety had a prevalence of 16.8%, 6.97%, and 5.96% among employees, respectively. The results showed that the prevalence of these disorders in employees with variable work shifts was higher than employees with fixed work shifts. With increasing age, disorders decrease such that older employees experience better mental health.^19^

 Research has shown that about 7 million people in Iran have some kind of mental disorder, and about 15-25% of the country’s population experience mild to severe depression, which is increasing due to social and environmental changes.^20^ Meanwhile, the knowledge of the people of most communities about factors related to depression, prevention approaches, self-care methods, and even treatment methods is very low.^21,22^ In this respect, almost half of the sufferers do not take any action regarding their treatment. Only 57% of employees with moderate depression and 40% with severe depression are treated to control depression symptoms.^23^

 Paying attention to mental health in all areas of life, including personal, social, and professional life, seems essential. At least one-third of people’s lives are spent in the workplace, and many of their social relationships are formed during office hours. Accordingly, it is necessary to maintain the mental health of employees in the first place as human beings and as people who are directly related to maintaining the health of other members of society.^14,23^ Based on the mentioned points, mental disorders strongly impact the productivity of individuals, organizations, and society’s economy. Besides, no study has been conducted to investigate the mental health status of the employees of Tabriz University of Medical Sciences. Therefore, the present study was carried out to determine the prevalence of psychiatric diseases among the employees of Tabriz University of Medical Sciences.

## Materials and Methods

###  Study Design and Setting

 The current study is a cross-sectional study carried out in 2019 in Tabriz. In this study, the data were obtained from the employees’ health cohort study of Tabriz University of Medical, which is part of the Persian Cohort study. The research population included the employees of Tabriz University of Medical Sciences who were selected and invited to participate in the study based on the inclusion criteria. The inclusion criteria were being employed as an official or contractual employee in the university headquarters of each cohort center, being over 18 years of age, and having Iranian citizenship. The study did not include people assigned to a service whose main place was in another city and employees with less than or equal to five years left until their retirement. Other exclusion criteria were incomplete answers to the questionnaire, being unable to answer, and reluctance to participate in the study. Considering the eligibility criteria, 1282 employees were included in the study. The participants were selected and invited by taking the list of people from the vice president for development. Each person was contacted by phone, the conditions of the study were explained to them, and those who were willing to participate in the study were selected. Employees who did not present themselves were contacted once or twice; if they did not wish to participate, they were not contacted again.

###  Measurements 

*The Composite International Diagnostic Interview (CIDI): *CIDI is a valid instrument to assess psychiatric disorders via interview. The first model of the CIDI was published in 1988 and has been updated periodically to mirror the changing diagnostic standards of DSM and ICD. This self-report instrument has 14 sections, each labeled with a letter (from A to X). It covers 17 major diagnostic domains and includes symptom-based questions to assess symptom severity and questions for clinical probing. Answers to the questionnaire items are Yes and No (yes = 5 and no = 1). In the present study, the questions of three sections of GAD (D_63_ to D_69_), major depressive disorder (MDD: E_1_ to E_54_), and obsessive-compulsive disorder (OCD: K_1_ to K_21_) were answered by the employees. This questionnaire is an instrument with acceptable validity and reliability as several studies have shown good to excellent reliability of CIDI across questioners and different cultures and times.^24,25^

###  Statistical Analysis

 Data were analyzed using the Stata statistical software version 17. Quantitative data were described with mean ± standard deviation (SD). Qualitative data were also described using frequency (percentage). In this study, psychiatric disorders (i.e. GAD, OCD, and MDD) were considered as dependent variables, and demographic variables (i.e. gender, age, education, marital status) as independent variables. The chi-square test was used to assess the prevalence of disorders among different categories of independent variables. Binary logistic regression was used to assess the association of independent and dependent variables by controlling potential confounding factors. *P* values < 0.05 were regarded as statistically significant.

## Results

 The present study was conducted at Tabriz University of Medical Sciences in 2019. The study population included 1282 university employees examined for psychiatric disorders, and most of them were women (54.06%). The average age of the study subjects was 41.7 (± 6.25) years. Also, 87.3% of them were married, and the educational level of most of them was bachelor’s degree (43.05%). Further information about the demographic characteristics of the participants is given in Table 1.

**Table 1:** Demographic Characteristics of Participants (N = 1282)

**Characteristics**		**Mean (SD)**
**Age**		41.7 (6.25)
		**N (%)**
Age groups (y)	20-30	55 (4.29)
	30-40	481 (37.52)
	40-50	641 (50)
	50-60	105 (8.19)
Gender	Men	589 (45.94)
	Women	693 (54.06)
Educational level	High school diploma	187 (14.59)
	Associate Degree	125 (9.75)
	Bachelor’s Degree	552 (43.05)
	Master’s Degree	396 (30.89)
	Doctoral Degree	22 (1.72)
Marital status	Single	119 (9.28)
	Married	1119 (87.29)
	Widowed	14 (1.09)
	Divorced	30 (2.34)
Cigarette smoking	Yes	81 (6.35)
	No	1193 (93.65)
Hookah use	Yes	38 (2.98)
	No	1236 (97.02)
Illegal drug use	Yes	1 (0.08)
	No	1273 (99.72)
Alcohol consumption	Yes	2 (0.16)
	No	1272 (99.84)

 According to the results of this study, 181 (14.12%) of the examined employees had GAD, 160 (12.48%) had MDD, and 50 (3.9%) had OCD (Table 2).

**Table 2:** Prevalence of Psychiatric Disorders among Employees of Tabriz University of Medical Sciences (N = 1282)

**Psychiatric Disorders**	**No. (%)**	**95% Confidence interval**	
		**Lower Limit**	**Upper Limit**
GAD	181 (14.12)	12.25	16.14
OCD	50 (3.9)	2.91	5.11
MDD	160 (12.48)	10.72	14.41

GAD, generalized anxiety disorder; OCD, obsessive-compulsive disorder; MDD, major depressive disorder.

**Figure 1:**
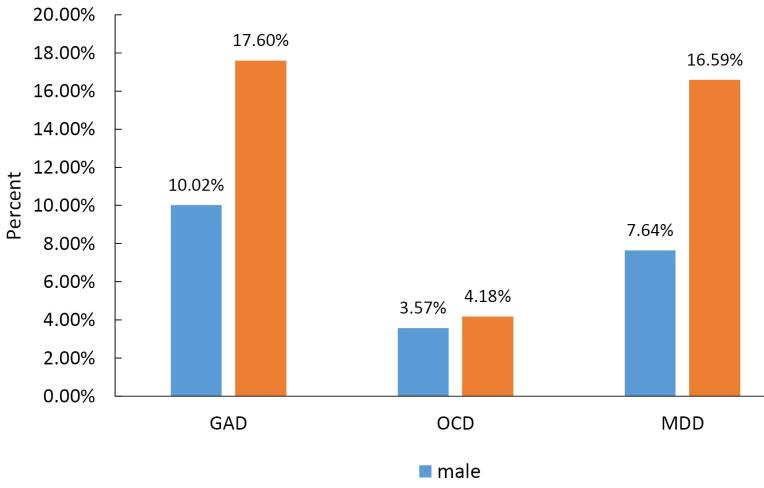
Prevalence of Psychiatric Disorders among Employees by Gender. GAD, generalized anxiety disorder; OCD, obsessive-compulsive disorder; MDD, major depressive disorder

 According to the chi-square test, the prevalence of GAD in women was significantly higher than men (17.6% in women and 10% in men) (Figure 1). A significant relationship was observed between the prevalence of GAD and marital status, such that the GAD prevalence was higher in widows than others (42.86%). There was no significant difference in terms of the prevalence of GAD in different age groups and education levels (Table 3).

**Table 3:** Prevalence of Psychiatric Disorders among Employees by Demographic Characteristics (Chi-square Test)

**Variables **		**GAD**	**OCD**	**MDD**
Gender	Male	59 (10.02)	21 (3.57)	45 (7.64)
	Female	122 (17.6)	29 (4.18)	115 (16.59)
*P* value		< 0.001	0.568	< 0.001
Age groups (y)	20-30	5 (9.09)	2 (3.64)	4 (7.27)
	30-40	67 (13.93)	20 (4.16)	55 (11.43)
	40-50	95 (14.82)	26 (4.06)	87 (13.57)
	50-60	14 (13.33)	2 (1.9)	14 (13.33)
*P* value		0.688	0.740	0.454
Education	High school diploma	29 (15.51)	8 (4.28)	16 (8.56)
	Associate Degree	21 (16.8)	2 (1.6)	14 (11.2)
	Bachelor’s Degree	85 (15.4)	29 (5.25)	90 (16.3)
	Master’s Degree	43 (10.86)	11 (2.78)	38 (9.6)
	Doctoral Degree	3 (13.64)	0 (0)	2 (9.09)
*P* value		0.262	0.149	0.009
Marital status	Single	19 (15.97)	4 (3.36)	16 (13.45)
	Married	151 (13.49)	42 (3.75)	134 (11.97)
	Widowed	6 (42.86)	2 (14.29)	3 (21.43)
	Divorced	5 (16.67)	2 (6.67)	7 (23.33)
*P* value		0.016	0.187	0.201

GAD, generalized anxiety disorder; OCD, obsessive-compulsive disorder; MDD, major depressive disorder.

 The MDD prevalence in women was significantly higher than men (16.59%). The maximum incidence of MDD was found amongst bachelors (16.3%), indicating a statistically significant difference. The prevalence of MDD in divorced people was higher than others (23.33%); however, the difference was not statistically significant. No significant difference was observed between the prevalence of MDD and age group. Further information about the prevalence of psychiatric disorders by demographic variables is given in Table 3.

 The association of psychiatric disorders with gender, age groups, education, and marital status was assessed using multiple binary logistic analyses (Table 4). Women had GAD significantly more than men [1.87 (1.33-2.65) *P* < 0.001]. Additionally, widowed individual had GAD significantly more than married people [4.06 (1.34-12.31) *P* = 0.013]. The results showed that women had MDD significantly more than men [2.18 (1.50-3.18) *P* < 0.001]. Individuals with a Bachelor’s degree had MDD significantly more than individuals with high school diploma [1.81 (1.02-3.22) *P* = 0.042].

**Table 4:** Binary Logistic Regression Model of Association of Psychiatric Disorders and Demographic Variables

**Variable**		**GAD**		**OCD**		**MDD**	
		**OR (95% CI)**	***P*** ** Value**	**OR (95% CI)**	***P*** ** Value**	**OR (95% CI)**	***P*** ** Value**
Gender	Men	Ref	Ref	Ref	Ref	Ref	Ref
	Women	1.87 (1.33-2.65)	< 0.001	1.05 (0.58-1.91)	0.849	2.18 (1.50-3.18)	< 0.001
Age groups	20-30	Ref	Ref	Ref	Ref	Ref	Ref
	30-40	1.69 (0.64-4.48)	0.284	1.15 (0.25-5.22)	0.849	1.67 (0.57-4.90)	0.348
	40-50	1.77 (0.68-4.63)	0.241	1.10 (0.24-4.90)	0.898	1.02 (0.69-5.86)	0.193
	50-60	1.45 (0.48-4.39)	0.506	0.46 (0.06-3.53)	0.456	2.02 (0.61-6.66)	0.245
Education	High school diploma	Ref	Ref	Ref	Ref	Ref	Ref
	Associate degree	1.15 (0.68-1.96)	0.590	2.75 (0.56-13.33)	0.209	1.23 (0.57-2.67)	0.588
	Bachelor’s degree	1.14 (0.70-1.83)	0.586	3.46 (0.81-14.73)	0.093	1.81 (1.02-3.22)	0.042
	Master’s degree	0.69 (0.46-1.02)	0.070	1.82 (0.39-8.35)	0.441	1.03 (0.55-1.93)	0.904
	Doctoral degree	1.08 (0.29-3.93)	0.904	Not included	Not included	1.24 (0.26-5.97)	0.781
Marital status	Single	1.17 (0.69-2.01)	0.544	1.13 (0.39-3.35)	0.816	1.03 (0.58-1.84)	0.899
	Married	Ref	Ref	Ref	Ref	Ref	Ref
	Widowed	4.06 (1.34-12.31)	0.013	6.12 (0.93-4.10)	0.059	1.71 (0.45-6.45)	0.426
	Divorced	1.1 (0.41-2.95)	0.850	2.02 (0.34-11.76)	0.442	1.83 (0.75-4.45)	0.177

GAD, generalized anxiety disorder; OCD, obsessive-compulsive disorder; MDD, major depressive disorder.

## Discussion

 This study was conducted to investigate the prevalence of psychiatric disorders among employees of Tabriz University of Medical Sciences. The results showed that 14.1% of employees had GAD, 12.5% had MDD, and 3.9% had OCD. Musarezaie et al indicated that the prevalence of depression among Isfahan University of Medical Sciences employees was 45.92%.^26^ The prevalence of depression among the employees of Zanjan University of Medical Sciences is reported to be 40%.^27^ In a study on 149 employees of Fasa University of Medical Sciences (Fars province, Iran), 38.6% of the employees suffered from various levels of depression. In this respect, 16.6%, 17.9%, and 4.1% of them had mild, moderate, and severe depression, respectively.^28^ In a study in Bangladesh in 2020 on healthcare workers, Tasnim et al reported the prevalence of anxiety at 41.2% and severe depression at 15.7%, which is almost in line with the present study’s findings.^29^ In the study by Acosta et al, the prevalence of depression among health system workers was 46%.^30^ In another study by Andrea et al on 3707 employees in the Netherlands, the 23-month cumulative prevalence of anxiety was 4.6% and depression was 3.3%.^31^ These differences in the prevalence of psychiatric disorders may be related to cultural differences, differences in systems, and differences in tools used to assess anxiety and depression. Besides, the results of the studies show that most employees are unaware of the symptoms of mental disorders, which can lead to underreporting the prevalence of psychiatric disorders. Providing necessary training to employees can help them recognize the signs and symptoms of anxiety and depression and encourage them to seek help from mental health professionals. Also, clinical screening of depression among employees by a qualified mental health specialist, followed by direct feedback and clinical referral, can be effective in the timely diagnosis of psychiatric disorders.^32^

 The results of the present study revealed that the prevalence of GAD in women was significantly higher than men. Also, there was a statistically significant relationship between gender and the prevalence of MD, such that depression was more common in women. However, there was no statistically significant relationship between gender and the prevalence of OCD. In line with the results of the present study, Musarezaie et al showed that the prevalence of MDD was higher in women than men.^26^ In the study by Rugulies et al in Denmark, the 5-year report of major depressive symptoms was 1.7% for men and 3.3% for women, which was higher in women.^33^ Tasnim et al investigated anxiety and depression among 803 people and reported its level to be higher in women; these results are in line with those of the current study.^29^ The higher prevalence in women compared to men is partly related to women’s susceptibility to psychiatric disorders, especially depression and anxiety disorders.^34^ In contrast, in the study by Andrea et al in the Netherlands, no difference was found between male (3.4%) and female (3.2%) employees in terms of the prevalence of depression.^31^

 The present study showed a significant relationship between the prevalence of depression and university employees’ education level. In this regard, the prevalence of MD increased from diploma to bachelor’s degree, while it decreased in master’s and doctoral degrees. In line with the results of the present study, Musarezaie et al reported a significant relationship between the prevalence of depression and the education level of university employees, such that the depression severity decreased with the increase in the education level.^26^ Dahlén and Janson also showed that the depression level declines with increasing education level.^35^ Elsewhere, Fallah et al indicated a significant relationship between depression and the education level of university employees.^27^ In a study conducted on 149 employees of Fasa University of Medical Sciences, there was a significant correlation between employee depression and their level of education.^28^ Generally, the results of these studies are in line with those of the present study, despite some contradictions in this field. For instance, in the present study, depression levels increased from a diploma to a bachelor’s degree and then decreased. Differences in data collection tools, sample size, and different inclusion and exclusion criteria in studies can be considered among the reasons for these contradictions.

 The present study showed a high prevalence of depression disorder in divorced people and a high prevalence of anxiety disorder in widowed people. Therefore, this category of people should be given more attention when providing mental health services to employees. In a study among nurses at Shiraz University of Medical Sciences, Hazhiri et al showed that single nurses have better mental health than married nurses.^36^ In comparison, in the present study, the prevalence of psychiatric disorders in married and single people was not significantly different.

 Garooci Farshi and Mani found a significant relationship between age and mental health in the dimensions of depression, obsession, and paranoia of employees such that with increasing age, the prevalence of disorders declines, and employees at older ages experience better mental health.^19^ However, the present study showed no significant relationship between age groups and the prevalence of psychiatric disorders.

 The current study is the first population-based study investigating the prevalence of psychiatric disorders among employees in Tabriz. Our study had some limitations. One of the limitations of the present study was that the research community was limited to the employees of Tabriz University of Medical Sciences, which makes it difficult to generalize this research to other university employees in the country. Another limitation was that not all psychiatric disorders were evaluated in this study. Also, medical records were not available to reveal the course of psychiatric disorders and treatment records. Investigating the factors determining anxiety and depression in university employees can provide valuable information in prevention planning at different levels, periodic examinations of employees, treatment, and prevention of psychiatric disorders for policymakers, planners, and managers of the country’s health and treatment systems.

## Conclusion

 The present study showed that anxiety and depression disorders in the employees of Tabriz University of Medical Sciences have a relatively high prevalence. Employees experience high stress and depression due to the nature of their job. Therefore, employers can create dedicated spaces for employees to rest and provide incentives to strengthen healthy behaviors to reduce employee stress. Also, strengthening counseling centers in the university, encouraging employees to participate in these centers, and examining them in terms of mental health will help identify people at risk of psychiatric disorders and provide counseling services to these people. Overall, addressing mental health issues in the work environment can reduce employee healthcare costs.
